# Characterization of Adenylyl Cyclase Isoform 6 Residues Interacting with Forskolin

**DOI:** 10.3390/biology12040572

**Published:** 2023-04-10

**Authors:** Vikram Bhatia, Saeid Maghsoudi, Martha Hinton, Anjali Y. Bhagirath, Nisha Singh, Appalaraju Jaggupilli, Prashen Chelikani, Shyamala Dakshinamurti

**Affiliations:** 1 Biology of Breathing Theme, Children’s Hospital Research Institute of Manitoba, Winnipeg, MB R3E 3P4, Canada; 2 Department of Oral Biology, University of Manitoba, Winnipeg, MB R3E 0W2, Canada; 3 Department of Pediatrics and Child Health, University of Manitoba, Winnipeg, MB R3A 1S1, Canada; 4 Department of Physiology and Pathophysiology, University of Manitoba, Winnipeg, MB R3E 0J9, Canada; 5 Department of Biochemistry and Medical Genetics, University of Manitoba, Winnipeg, MB R3E 0J9, Canada

**Keywords:** adenylyl cyclase, cyclic AMP, homology modeling, forskolin, site-directed mutagenesis

## Abstract

**Simple Summary:**

Adenylyl cyclase isoform 6 is the only member of the adenylyl cyclase enzyme family to be inhibited by low oxygen levels. We studied the unique structure of the adenylyl cyclase 6 molecule, using computer models that draw upon known adenylyl cyclase structures. We also mutated several amino acids in that structure, to understand which of the amino acids in adenylyl cyclase 6 interact with the plant compound forskolin to activate this enzyme. We hope to use this knowledge to design a selective activator of adenylyl cyclase 6.

**Abstract:**

Background: The adenylyl cyclase (AC) pathway, crucial for pulmonary vasodilation, is inhibited by hypoxia. Forskolin (FSK) binds allosterically to AC, stimulating ATP catalysis. As AC6 is the primary AC isoform in the pulmonary artery, selective reactivation of AC6 could provide targeted reinstatement of hypoxic AC activity. This requires elucidation of the FSK binding site in AC6. Methods: HEK293T cells stably overexpressing AC 5, 6, or 7 were incubated in normoxia (21% O_2_) or hypoxia (10% O_2_) or exposed to s-nitrosocysteine (CSNO). AC activity was measured using terbium norfloxacin assay; AC6 structure built by homology modeling; ligand docking to examine FSK-interacting amino acids; roles of selected residues determined by site-directed mutagenesis; FSK-dependent cAMP generation measured in wild-type and FSK-site mutants by biosensor-based live cell assay. Results: Only AC6 is inhibited by hypoxia and nitrosylation. Homology modeling and docking revealed residues T500, N503, and S1035 interacting with FSK. Mutation of T500, N503, or S1035 decreased FSK-stimulated AC activity. FSK site mutants were not further inhibited by hypoxia or CSNO; however, mutation of any of these residues prevented AC6 activation by FSK following hypoxia or CSNO treatment. Conclusions: FSK-interacting amino acids are not involved in the hypoxic inhibition mechanism. This study provides direction to design FSK derivatives for selective activation of hypoxic AC6.

## 1. Introduction

Cyclic adenosine 3′,5′-monophosphate (cAMP), the first identified intracellular second messenger, plays a pivotal role in a wide range of regulatory mechanisms triggered by extracellular signal transduction [1,2]. Adenylyl cyclases (ACs), widely expressed in prokaryotic and eukaryotic cells, are the effector enzymes that generate cAMP from cytosolic adenosine triphosphate (ATP) and regulate several vital cellular processes. Mammalian cells express nine membrane-bound AC isoforms (mACs)—AC1 to AC9, and one soluble AC (sAC, AC10) with no transmembrane (TM) domains [3,4].

All mACs share architectural resemblance based on their protein sequences. They are membrane proteins with two membrane-bound regions, each with six TM domains and two helices that link TM6 and TM12 to pseudo-symmetric cytosolic catalytic domains 1 (C_1_) and 2 (C_2_) [5,6] with amino (N-) and carboxyl (C-) terminals. Both terminal portions are highly variable, whereas the catalytic domains are mostly conserved [7,8]. The C_1_ and C_2_ domains are interconnected and constitute the interface of active and allosteric binding sites [9]. The catalytic moiety and a P-loop accommodate the substrate ATP at the active binding site. In contrast, the allosteric site forms a pocket for forskolin (FSK), which activates the AC enzyme [10,11]. Based on their patterns of homology and functioning, the mACs are further divided into four groups; Ca^++^-stimulated group I (AC1, AC3, and AC8), Gβγ-stimulated group II; (AC2, AC4, and AC7), Gαi/Ca^++^-inhibited group III (AC5 and AC6), and the forskolin-insensitive group IV (AC9) [3,12].

Cellular and systemic oxygen homeostasis is an intricately regulated process for metabolism, protein synthesis, and cell survival. Hypoxia is defined as insufficient tissue oxygen for physiological function [13]. We have previously shown that neonatal hypoxia ablates the pulmonary arterial cAMP response to prostacyclin receptor stimulation [14]. Prolonged hypoxia selectively reduces Gαs levels and reduces β2-adrenergic signaling to cAMP [15,16]. We previously showed that pulmonary artery myocytes express AC6 predominantly, followed by isoforms 3, 7, and 9. In hypoxic pulmonary hypertension, AC activity is decreased by hypoxia and by nitric oxide, as a result of protein nitrosylation [17]. Sensitivity to hypoxia is not uniform among AC isoforms. AC6 and 7 both contain hypoxia inducible factor (HIF)-response elements in their promoters, upregulating their expression in hypoxia [18]. AC6 is critical for cardiac function, and the principal vasodilator in vascular myocytes [19], particularly in fetal and neonatal tissues [20]. The catalytic activity of AC6 but not AC2 or 5 is attenuated by NO [21], suggesting differential regulation by nitrosylation. Although AC6 and AC5 share 91.5% sequence alignment [22] and both have decreased expression in heart failure [23], AC5 disruption is cardio-protective [24], whereas AC6 overexpression is protective in cardiac injury [25]. Therefore, in this study, we compare sensitivities of AC 5, 6, and 7 to inhibition by hypoxia or by nitrosylation.

Our lab’s previous findings suggest that selective reactivation of AC6 might provide a targeted approach to restore hypoxic AC activity. Forskolin, a plant diterpene from the plant *Coleus forskolhii*, is a direct, reversible but non-selective activator for AC isoforms. Most mACs can be activated by FSK and inhibited by adenosine analogs known as P-site inhibitors [26,27,28]. Virtual ligand screening studies showed colforsin, a commercially available FSK derivative, as a semi-selective activator of AC6; the development of derivatives with greater isoform selectivity will require a greater structural understanding of the AC6 FSK binding pocket. The initial structures of mACs were the homodimer of the C_2a_ catalytic domain of the AC2 (Rat) [29] and the pseudo-dimer of the C_1a_ domain of the canine AC5 [9]. The cryo-electron microscope structure of the bovine AC9–Gαs complex was recently reported as a full-length AC9 isoform with TM and catalytic domains [30]. Crystal structures of AC5C_1_/AC2C_2_/Gαs (PDB ID: 1U0H) were used to guide model building for the AC9-Gαs [31]. We previously reported an in-silico model of the 3D structure of AC6 catalytic domains using PDB: 1CJK (containing C_1_ domain from canine AC5, C_2_ from rat AC2, and bovine Gαs) as a homology template for the AC6 protein sequence [22]. However, that study provided no information on TM domains. On the other hand, the full-length AC9 cryo-EM structure cannot fully define the AC6 FSK binding site, as AC9 is considered relatively FSK-insensitive.

In the current study, we use the AC9 cryoEM structure as a template to build a full-length AC6 three-dimensional (3D) structure using the homology modeling approach. We predict the isoform-specific residues involved in AC6 interactions with ligands ATP and FSK by docking these ligands and analyzing interacting residues, including conserved residues at the ATP binding site (E386, F388, R1146, K1152). To validate the structure, we use model-guided site-directed mutagenesis of selected residues at the allosteric activator (FSK) binding site (T500, N503, S1035) in a structure-function approach [32,33]. We also examine the hypoxia sensitivity of AC6 FSK site mutants, to determine if any residues at the FSK site may be involved in hypoxia-mediated AC inhibition. Our goal here was to ascertain whether the FSK site of AC6 is a credible target for drug design, to achieve restoration of hypoxic inhibition of catalytic activity.

## 2. Materials and Methods

### 2.1. Materials

Human embryonic kidney (HEK) 293T cells were purchased from American Type Culture Collection (Manassas, VA, USA) and maintained in a cell-culture medium composed of DMEM-F12 (Thermo Fisher Scientific, Carlsbad, CA, USA) supplemented with 10% heat-inactivated fetal bovine serum (MilliporeSigma, Burlington, MA, USA) and 1% penicillin-streptomycin (100 mg/mL) (Thermo Fisher Scientific, Carlsbad, CA, USA). Hygromycin (200 μg/mL; Sigma-Aldrich, Mannheim, Germany) was used in the selection medium to generate stable cell lines. All chemicals were of analytical grade. The cAMP real time assay kit (cADDis) was obtained from Montana Molecular (Bozeman, MT, USA). Forskolin and IBMX were purchased from Sigma Aldrich, Canada. HRP-conjugated mouse monoclonal anti-FLAG antibody was purchased from Abcam; anti β-actin mouse monoclonal antibody from Sigma-Aldrich (St. Louis, MO, USA).

### 2.2. Molecular Biology and Cell Culture

The N-terminal FLAG epitope-tagged human AC isoform 6 (AC6) gene and corresponding site-directed mutants at the ATP and FSK binding interface of AC6 were codon-optimized for expression in mammalian cells and cloned into the KpnI-NotI restriction site of the pcDNA 3.1/Hygro (+) expression vector. The wild-type (WT) and corresponding mutants of AC6 carried by expression vector pcDNA 3.1/Hygro (+) were commercially synthesized by GenScript (Piscataway, NJ, USA). Per the manufacturer’s instructions, the gene construct of AC6 WT and mutants were transiently expressed in HEK293T cells using Lipofectamine 2000 (Thermo Fisher Scientific, Waltham, MA, USA). We generated stable HEK293T cells expressing the WT and mutants in the selection medium containing 200 µg/mL hygromycin as described previously in references [34]. The stable cell lines used in the study were characterized using Western blot analysis and flow cytometry.

### 2.3. Adenylyl Cyclase Activity Assay

AC6 wild type, FSK-site mutants, and naïve HEK293T cells were grown in normoxic, hypoxic, and nitrosyl group donor CSNO treatment for 30 min [17,35]. Cells were collected and lysed in 20 mM Tris buffer pH 7.4 with protease inhibitors. Protein content was estimated in lysates by using the Bradford method and adjusted to 5 µg protein/µL. The AC activity assay was done in 96-black-well plates, started by addition of 50 µL lysate to wells already containing 1 mM ATP, 0.25 mM Terbium-III, 0.05 mM Norfloxacin, 10 mM MgCl_2_, 20 µM CaCl_2_, 20 mM Tris-HCl, and 1% bovine serum albumin, at 37 °C. ATP dose-response (10^−6^ to 10^−2^ M) and FSK dose-response (10^−7^ to 10^−5^ M) effects on AC activity were also determined in a similar manner. The fluorescence intensity of terbium-norfloxacin was acquired by FlexStation3 (Molecular Devices; 337 nm excitation, 545 nm emission), and the loss of ATP-bound terbium norfloxacin fluorescence due to ATP-to-cAMP conversion was calculated [36], with AC specific activity determined as a substrate concentration function, as Δfluorescence/min/mg protein. Experiments were performed per lysate in triplicate and three independent biological replicates.

### 2.4. Western Blot Analysis

20 µg of whole cell extracts were separated on SDS-PAGE and transferred on a nitrocellulose membrane using a transfer apparatus according to the manufacturer’s protocols (Bio-Rad, ON, Canada). For detection of FLAG-tagged AC isoforms, after transferring, the membranes were first blocked by incubation with 3% fish gelatin prepared TBST (10 mM Tris, pH 8.0, 150 mM NaCl, 0.5% Tween 20) for 60 min, and the membrane was incubated with antibodies against anti-FLAG (1:1000), β-actin (1:10,000) at 4 °C for 12 h. Membranes were washed thrice with TBST for 10 min and for β-actin (Invitrogen, ON, Canada), incubated with a 1:5000 dilution of horseradish peroxidase-conjugated anti-mouse (Bio-Rad) for 2 h. Anti-FLAG was HRP conjugated (ab49763, Abcam, Cambridge, MA, USA). Blots were washed with TBST three times and developed with the clarity max ECL system (Bio-Rad) according to the manufacturer’s instructions.

### 2.5. Molecular Modeling and Ligand Docking

The modeling and docking protocols were followed as described previously [37,38]. The 3D homology model for human AC6 was built using AC9 cryoEM (PDB ID: 6R4O) structure as a template [30] based on the homology modeling approach (Schrodinger Maestro v12.2 suite, New York, NY, USA). The AC6 sequence was submitted to the I-TASSER server to check other templates. The server gives ten templates based on sequence similarity and follows the threading of 3D models; 6R4O was among the optimal models. The AC9 3D structure was used as a homology template, as the first AC structure reported with complete transmembrane and catalytic domains, though the homology between human AC6 and AC9 sequences is 23.9%. The loops of side chains of the AC6 structure were refined, and the model was energy minimized by performing 1000 steps of gradient algorithms using the PRIME model of Schrodinger software. The quality of the model was checked using Procheck (https://servicesn.mbi.ucla.edu/PROCHECK/ accessed on 10 February 2020). A Ramachandran plot showed >98% of residues were in favorable and allowed regions.

After the quality check, the AC6 model was used to dock ATP at the catalytic site and FSK at the allosteric site at the catalytic domains interface of AC6. Briefly, the AC6 model was prepared for ligand docking by the protein preparation wizard. A grid area was generated around the catalytic domain 1 and 2 for ligand docking by selecting amino acid residues and their location in the AC6 sequence. The selected amino acids are conserved and were reported previously in other isoforms of AC, like AC9, as involved in the interaction with ATP and FSK. The 2-dimensional (2D) chemical structures of ATP and FSK were obtained from the PubChem database, and further, their 3D structures were optimized using the Ligprep module in Schrodinger. The optimized ligands were docked into the AC6 protein structure using the Glide (extra precision XP) module. Based on the Glide score, the best poses were selected, and the complex was further energy minimized using the steepest descent and conjugate gradient algorithms. The energy-minimized and stimulating complex of AC6 docked with both ligands was analyzed to study the interactions. The PyMol molecular visualization software was used to analyze the structural interactions between ligands and the respective AC6 model.

### 2.6. Amino Acid Sequence Analysis and Selected Mutants

The amino acid sequences of all nine human AC isoforms (AC 1–9) were retrieved from the UniProt database (https://www.uniprot.org/, accessed on 10 February 2020). Multiple sequence alignment was performed using the Clustal omega multiple sequence alignment algorithms. All selected conserved residues at close proximity to ATP and FSK binding domains were identified.

### 2.7. cAMP Real-Time Measurement Assay

For real-time measurement of cAMP generation in live HEK293T cells stably expressing AC6 and FSK binding mutants, subconfluent cells plated on a black-walled, clear, flat-bottomed 96 well plate (Greiner Bio-One, Monroe, NC, USA) treated with poly-l-lysine with 100 µL of cell media (50,000 cells/well). 40 µL of baculovirus modified with a mammalian promoter expressing the green cAMP difference detector in situ (cADDis) cAMP sensor (Montana Molecular) and 2 mM sodium butyrate per well [39,40]. Cells were grown overnight at 5% CO_2_ and 37 °C. Media were aspirated and replaced with 150 µL per well of Dulbecco’s PBS solution without calcium and magnesium. The 96-well plate was covered with aluminum foil and incubated at room temperature for 30 min. Cell fluorescence was read bottom using excitation/emission wavelengths of 488/20 nm and 525/20 nm, respectively, with a FlexStation3 plate reader (Molecular Devices). A 10-min kinetic read on unstimulated cells was performed to normalize each well’s fluorescence variability. Cells were stimulated with forskolin in a dose-dependent manner (0.621–10 µM), and fluorescence changes in each well were read at 30-s intervals for 40 min. Data were fit to a single-site decay model using Prism8.0 software (GraphPad Software Inc., San Diego, CA, USA). The kinetic rate constant (k) was compared between wild type and mutants by constraining the curve fits to a common plateau and plotting k to compare the rate of cAMP production across a different dose of forskolin. Different doses of forskolin were prepared in separate 96 well plates and used machine dispenser to add doses in corresponding wells. 3-isobutyl-1-methylxanthine (IBMX) was added as a PDE inhibitor, and β2-AR with isoproterenol response was used as a positive control of the assay [41].

### 2.8. Statistical Analysis

Statistical analyses were performed with GraphPad Prism v.8.0 software (GraphPad Software Inc., San Diego, CA, USA), using an unpaired Student’s *t*-test or one-way ANOVA with Tukey’s post hoc test and a minimum of 3–5 independent experiments to determine statistical significance; * *p* < 0.05, ** *p* < 0.01, *** *p* < 0.001.

## 3. Results

### 3.1. Basal and Hypoxic Catalytic Activity in AC Isoforms

**Figure 1:**
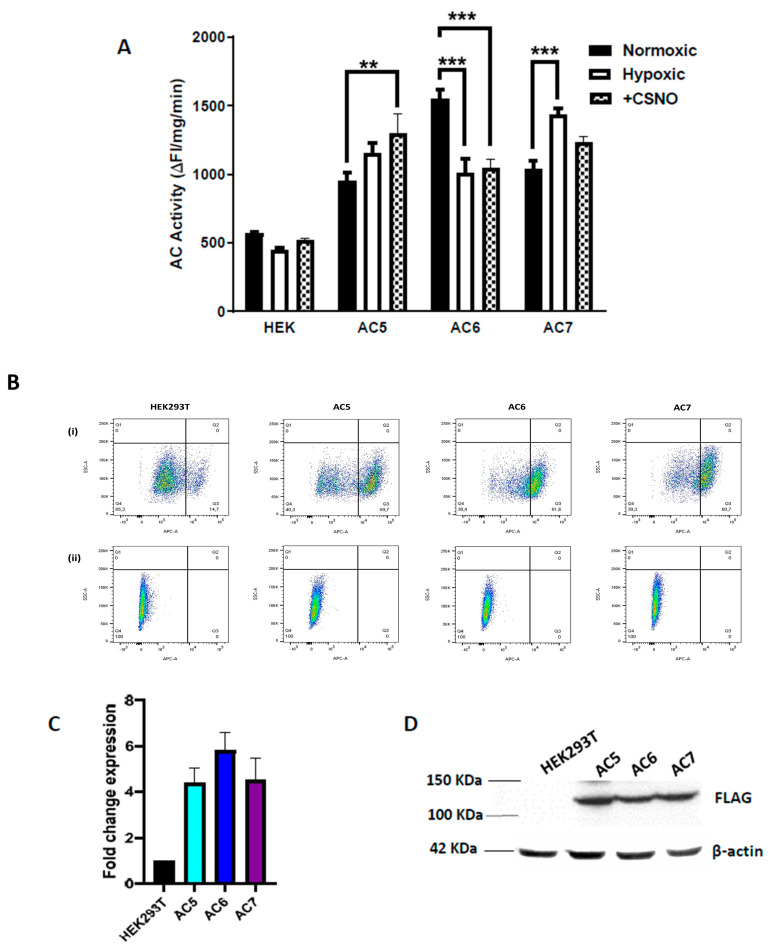
AC activity in naïve HEK293T cells and AC isoforms 5, 6 or 7 stably overexpressed in HEK293T, exposed to (**A**) 72 h hypoxia (10% O_2_, 5% CO_2_) or 30 min 250 µM nitrosocysteine (CSNO) treatment. Only isoform AC6 is inhibited by hypoxia and by CSNO treatment. N = 3, analysis by two-way ANOVA, ** *p* < 0.01, *** *p* < 0.001. (**B**) Expression of AC isoforms 5, 6, and 7 quantified by flow cytometry of cells fixed with 4% paraformaldehyde and permeabilized with 1% saponin in FACS buffer, using (**i**) APC-conjugated mouse monoclonal anti-FLAG (DYKDDDDK) antibody (quadrant Q3 shows APC positive expression), and (**ii**) data normalized against mouse monoclonal IgG antibody; analyzed using FlowJo™ v10.8 Software (BD Life Sciences, Ashland, USA). (**C**) Fold change in expression by flow cytometry, calculated as APC-FLAG positive cells. Data indicate fold change ± SD, obtained from at least three independent experiments. (**D**) Western blot of FLAG indicating AC5, AC6, and AC7 expression in 20 μg protein from HEK293T whole cell lysates separated on 10% SDS-PAGE, probed with mouse monoclonal HRP-anti-FLAG antibody (1:1000, Abcam, Cambridge, MA, USA). HEK293T cells not expressing a FLAG-tagged protein used as antibody control; β-actin as the loading control.

AC5 and AC6 are the closest isomers with sharing high protein sequence similarities, while AC6 and AC7 isoforms both contain hypoxia-inducible factor-response elements in their promoter regions. We generated AC5, 6, and 7 isoform overexpression cell lines in HEK293T cells (expressed without their respective promoters), and examined the effect of hypoxia and normoxia on AC catalytic activity (Figure 1A). AC6 showed a significant loss of basal catalytic activity following 72 h hypoxia (10% O_2_), whereas AC7 basal activity was elevated in hypoxia compared to normoxia (21% O_2_). AC5 activity was not significantly altered by hypoxia.

Using nitrosocysteine (250 µM CSNO, a nitrosyl group donor), we examined the effect of nitrosylating stress on the catalytic activity of the selected AC isoforms and found that AC6 activity also significantly decreased with CSNO treatment (Figure 1A) compared to control, whereas AC 5 and 7 showed increased AC activity.

The gene constructs used for all isoforms have a FLAG tag at the intracellular N-terminus, which allowed detection by an anti-FLAG antibody. Cell surface expression of AC isoforms was estimated by flow cytometry quantification of AC isoform expression (Figure 1B,C), using a limited membrane permeabilization protocol for FLAG immunostaining to identify tagged proteins within the submembrane space. In whole cell lysates in stable cell lines, Western blot also showed similar expression of all isoforms (Figure 1D). Untransfected HEK293T was used as negative control having no FLAG expression, and β-actin was used as a loading control.

### 3.2. Molecular Modeling and Ligand Docking of AC6

**Figure 2:**
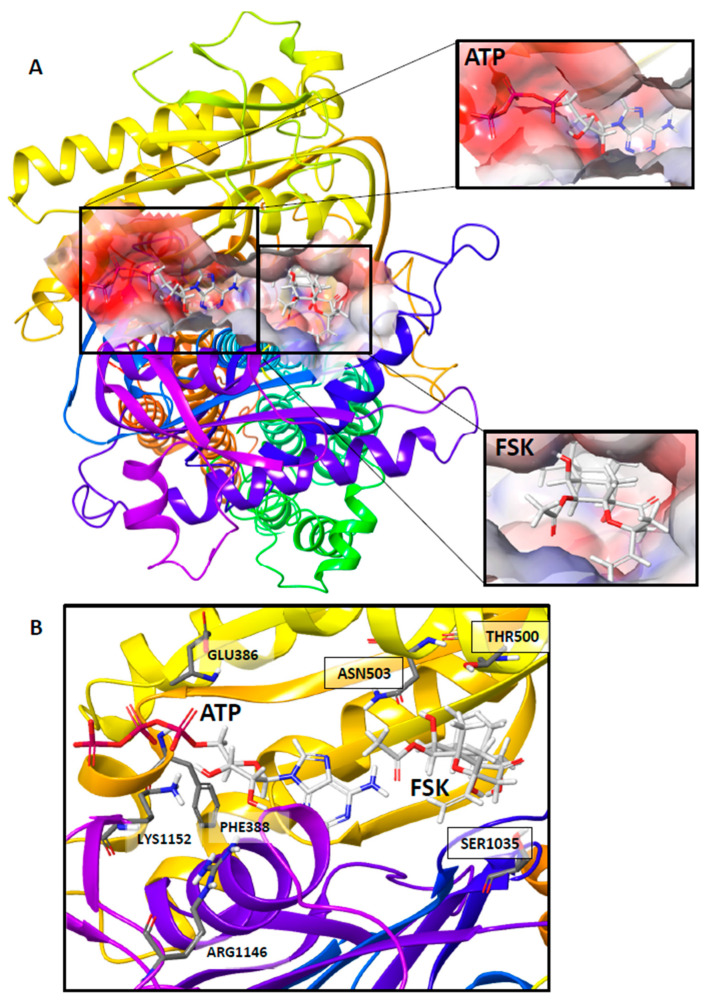
Molecular model of human adenylyl cyclase isoform 6 bound to adenosine triphosphate (ATP) and Forskolin (FSK). The homology model of human AC6 was built using Schrodinger Maestro v12.2, including catalytic domain C_1_, and domain C_2_ with transmembrane helix (TM). (**A**) FSK (lower subimage) and ATP (upper subimage) are bound in the interface between AC6-C1 and AC6-C2. The blown-out image of the binding site surface shows the docked ATP at the catalytic site (upper callout) and FSK at the allosteric site (lower callout) of the 3D molecular structure of AC6. (**B**) Residues Glutamate (GLU386), Phenylalanine (PHE388), Arginine (ARG1146), and Lysine (LYS1152) appear in close proximity to the ATP binding site; Threonine (THR500), Asparagine (ASN503), and Serine (SER1035) at the FSK binding site. The latter were mutated to validate the AC6 molecular structure.

As the catalytic activity of the AC6 isoform was uniquely attenuated by hypoxia and CSNO treatment, we felt a valid AC6 structure may help in the design of potential selective activators of AC6. To understand the structure of the AC6 isoform, since the crystal structure of any human AC is unavailable from the RCSB protein databank, we employed a knowledge-based homology modeling approach to predict the 3D structure of AC6 using the Maestro module of Schrodinger. AC9 isoform cryo-EM 3D structures were reported as PDB: 6R3Q, 6R4O, 6R4P. 6R3Q was the structure of a membrane adenylyl cyclase bound to an activated stimulatory G protein; whereas 6R4O has a truncated sequence, and 6R4P is a structure of only the soluble domain of adenylyl cyclase. Thus, we used 6R3Q as a template to build the AC6 model with complete transmembrane and cytosolic catalytic domains (Figure 2A). The residues involved in MANT-GTP interaction, an ATP analog, and FSK interaction with AC9 (PDB: 6R4O) were copied to dock both ATP and FSK ligands to the AC6 model. We used the Ligdock and Glide module of the Schrodinger platform for docking with 25 different poses in the binding pocket. The pose with the highest glide score was selected and grouped with the molecular model of AC6. The structure was further energy minimized and considered for interacting residues analysis. The glide score indicates the binding energy based on algorithms. The higher the score better the binding. Even though no score function is completely accurate, we analyzed the ligand’s orientations also in the binding pocket and compared them with our previous AC6 model.

To generate the grid for ATP docking to the AC6 catalytic pocket, we selected the following residues: D384, I385, E386, G387, F388, T389, F408, A409, R410, I425, L426, G427, and D428 from the C_1_ domain; and K1031, D1105, I1106, and G1108. N1109 and K1152 from the C_2_ domain. Subsequently, V499, T500, and N503 from the C_1_ domain and I1033, G1034, S1035, and T1036 from domain C_2_ were picked for receptor grid preparation to dock FSK at the allosteric site.

**Figure 3:**
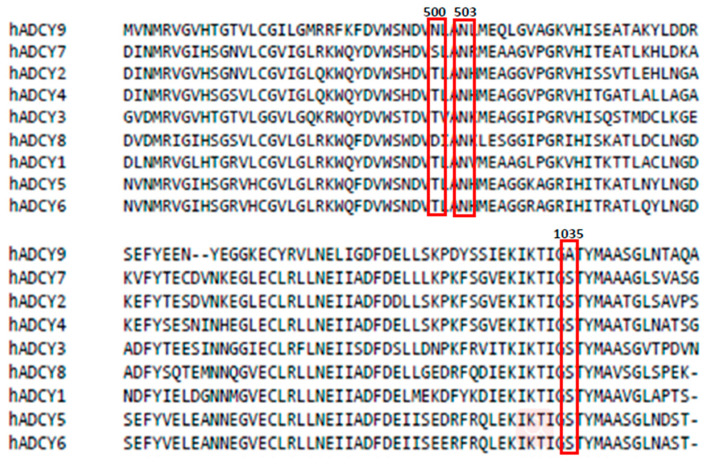
Multiple sequence alignment of all 9 human AC isoforms protein sequences. The amino acid sequences were retrieved from the UniProt database, and sequence alignment was performed using Clustal omega. The amino acid residues selected for mutational studies are highlighted in the red bracket. Amino acids selected for mutation in the AC6 isoform are conserved in all human adenylate cyclase isoforms, except Threonine (T500) in catalytic domain C1.

Residues E386 and F388 in the C_1_ domain and R1146 and K1152 in the C_2_ domain directly interact with substrate ATP at the catalytic binding site, with hydrogen bonding (Figure 2B). Residues T500 and N503 in C_1_, and S1035 in C_2_ are involved in interaction with ligand FSK at the allosteric binding site. All these residues are in close proximity to both ligand binding sites, and except E386 and S1035, are conserved in all the AC isoforms. E386 is conserved only in AC5 and 6 isoforms, whereas S1035 is aligned with the alanine residue of AC9 (Figure 3). Our in-silico findings of specific residue interactions with ATP were similar to that reported in previous studies.

We then performed site-directed mutagenesis to validate the AC6 molecular model and examine the interacting residues’ significance, mutating all FSK-interacting residues to alanine. Mutational modeling analysis was performed to predict structural changes in mutants compared to the AC6 wild type (WT). The mutant structures were energy minimized and superimposed with the AC6 WT structure. We did not see notable deviations (<1 Å) in orientation of docked ligands when we superimposed the 3D structure of each mutant with WT structure.

**Figure 4:**
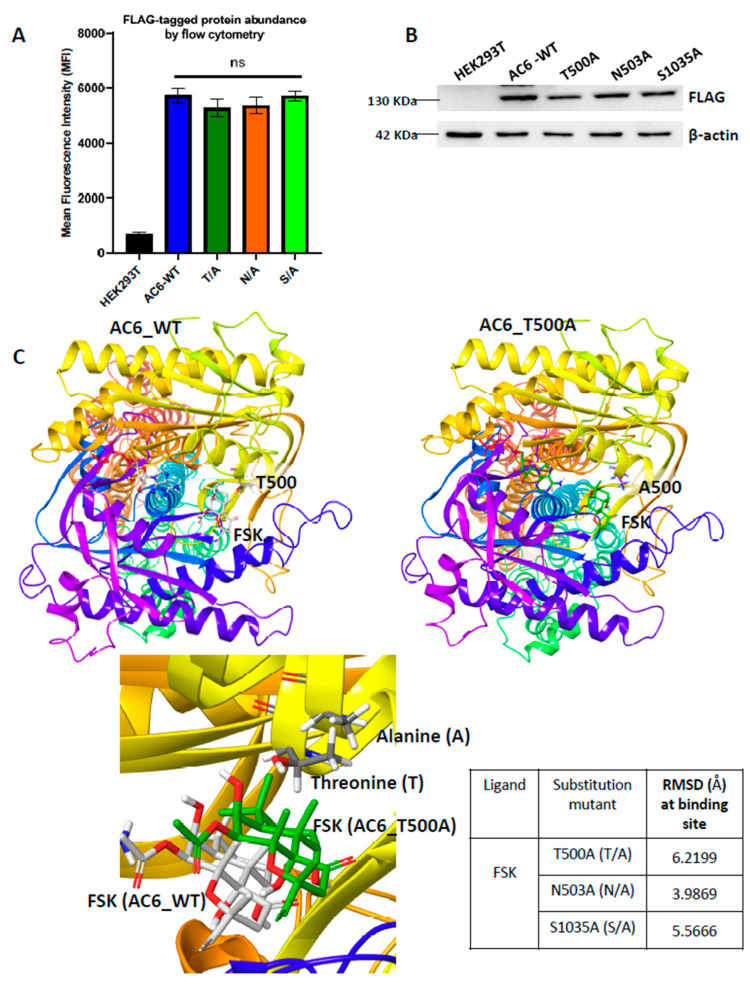
(**A**) Expression of AC6-WT and FSK- binding site mutants (T500A, N503A, and S1035A) quantified by flow cytometry of HEK293T cells fixed with 4% paraformaldehyde and permeabilized with 1% saponin in FACS buffer, using APC conjugated mouse monoclonal anti-FLAG antibody, normalized against mouse monoclonal IgG antibody, analyzed using FlowJo™ v10.8 software and represented as fold change from untransfected HEK293T. Data indicate mean ± SEM obtained from at least three independent experiments, analyzed by one-way ANOVA with Dunnet’s post hoc test for multiple comparisons, differences were non-significant (ns). (**B**) Western blot of FLAG-tagged AC6 WT and mutants T500A, N503A, and S1035A in 20 μg protein in total cell lysates separated on 10% SDS-PAGE and probed with mouse monoclonal HRP-anti-FLAG antibody (1:1000, Abcam). (**C**) T500A structure generated using a mutational modeling approach (right), energy minimized and superimposed on AC6-WT structure (left) to observe conformational deviations (lower panel). Root mean square deviation (RMSD) at the FSK binding site was calculated for all three substitution mutants versus AC6 WT using the superposition tool of Maestro v20.4 software (Schrödinger), showing the highest RMSD value generated among FSK binding site residues of the T/A mutant.

In contrast, there was a substantial rearrangement in the residues involved in interacting with FSK at binding pockets following alanine substitution. The superimposed mutant AC6-T500A and WT model showed the highest root mean square deviation (RMSD) of ~6.21 Å (Figure 4C).

We carried out a preliminary screening process for FSK-interacting residues using transient transfections with alanine substitution mutants, and FSK response measured by live cell cAMP assay, to select positive residues for subsequent stable transfection and detailed investigation. For example, Val499 mutated to alanine resulted in no observed difference in FSK response versus WT AC6, while Ser1035 mutated to alanine inhibited the FSK response (Supplemental Figure S1). Protein expression levels could not be well controlled by transient transfection, so this was used only as an initial tool to select pertinent residues. 

### 3.3. Site-Directed Mutagenesis and AC6 Activity

To validate the AC6 structure, a molecular model-guided mutagenesis approach was followed, where we selected the residues T500, N503, and S1035 involved in FSK interaction with AC6. Residues were mutated to alanine, a smaller amino acid that is not amenable to nitrosylation and would have minimal effects on protein folding and ligand binding (Figure 2B). Substitution mutant T500A is abbreviated hereafter as T/A, N503A as N/A, and S1035A as S/A.

**Figure 5:**
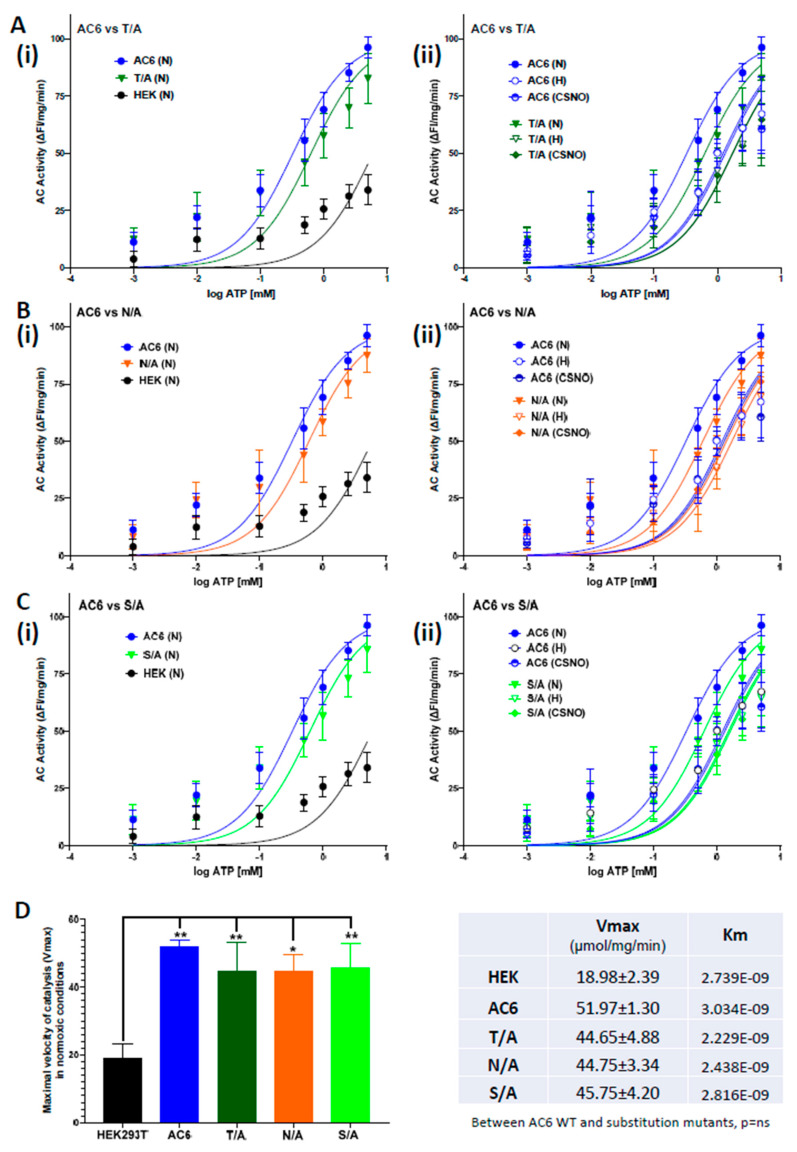
ATP dose-dependent catalytic activity in AC6 Forskolin binding site mutants. AC activity is measured as a loss of luminescence (FI) of terbium norfloxacin when unbound from ATP (substrate) and bound to inorganic phosphate donated by the action of AC. AC activity vs ATP substrate concentration (10^−6^–10^−2^ M) in (**A**) AC6 WT vs. mutant T/A; (**B**) AC6 WT vs. mutant N/A; and (**C**) AC6 WT vs. S/A was (**i**) quantified in normoxia (N, 21% O_2_, 5% CO_2_), and (**ii**) compared with hypoxia (H, 10% O_2_, 5% CO_2_) or with 250 µM nitrosocysteine (CSNO, 30 min) treatments. The maximum velocity of catalysis (Vmax) and Michaelis constant (Km, equivalent to the concentration that provides 50% of Vmax) of ATP-mediated AC activation was calculated by a nonlinear regression curve lit using Michaelis-Menten equation. All FSK binding site mutants exhibited comparable catalytic activity, with catalytic velocities close to that of WT AC6 under normoxic conditions (**D**). Differences in Vmax and Km values between WT and mutant AC6 in normoxic conditions analyzed by one-way ANOVA analysis indicated nonsignificance. WT AC6 Vmax was significantly higher than that of naïve HEK293T, confirming AC6 overexpression. N = 3, * *p* < 0.05, ** *p* < 0.01, ΔFI, change in fluorescence intensity.

Multiple sequence alignment of all AC isoforms showed N503 is conserved in all AC isoforms and S1035 is conserved, except in AC9, and T500 is conserved in all but AC9 and AC8 (Figure 3). All the mutant constructs were stably expressed in HEK293T cells using hygromycin selection. Expression levels were verified by flow cytometry (Figure 4A) and by Western blot of whole cell lysates (Figure 4B). To confirm the effect of these mutations on AC6 catalytic activity, AC-specific activity versus ATP substrate was determined in a dose-dependent manner (Figure 5A–C); maximal velocity of catalysis (Vmax) was calculated by nonlinear regression curve fit and Michaelis-Menten equation (Figure 5D). All mutants tested were equally capable of ATP-stimulated catalytic activity as AC6 WT (Figure 5(Ai–Ci). Under either hypoxia or CSNO treatments, Vmax decreased in AC6 WT; the same phenomena were observed in all three substitution mutants (Figure 5(Aii–Cii). No significant differences in the Michaelis-Menten catalytic constant (Km) were observed (Figure 5D).

**Figure 6:**
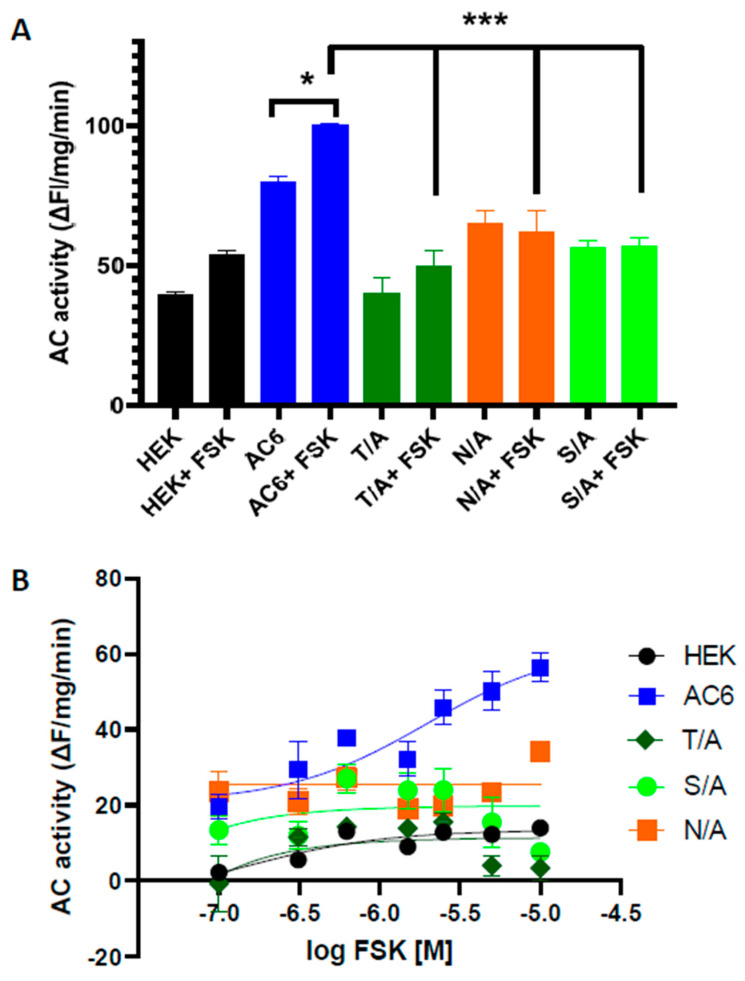
Effect of site-directed mutagenesis at FSK allosteric site on AC activity in response to forskolin challenge. Basal and stimulated (10 µM FSK) AC activity (**A**), and the FSK-stimulated dose response (**B**) were measured in the total cell lysate of naïve HEK293T cells, AC6 WT, and T500A, N503A or S1035A mutants of forskolin interacting residues, in the presence of 1 mM ATP substrate. N = 3, * *p* < 0.05, *** *p* < 0.001.

We then examined the responsiveness of the FSK site mutants versus AC6 WT to stimulation with FSK. Unstimulated AC enzymatic activity was decreased in all the mutants compared to AC6 WT when captured at a single submaximal (1 mM) concentration of ATP substrate (Figure 6A). Only AC6 WT showed a significant increase in activity when stimulated with FSK; there were no significant increases in activity of FSK site mutants in response to the FSK challenge. While all mutants resulted in significantly lowered FSK-stimulated AC activity, the T/A mutation appeared the most impaired (Figure 6A). The forskolin dose-response study showed cAMP generation in response to FSK was impaired in all mutants compared to AC6 WT (Figure 6B).

**Figure 7:**
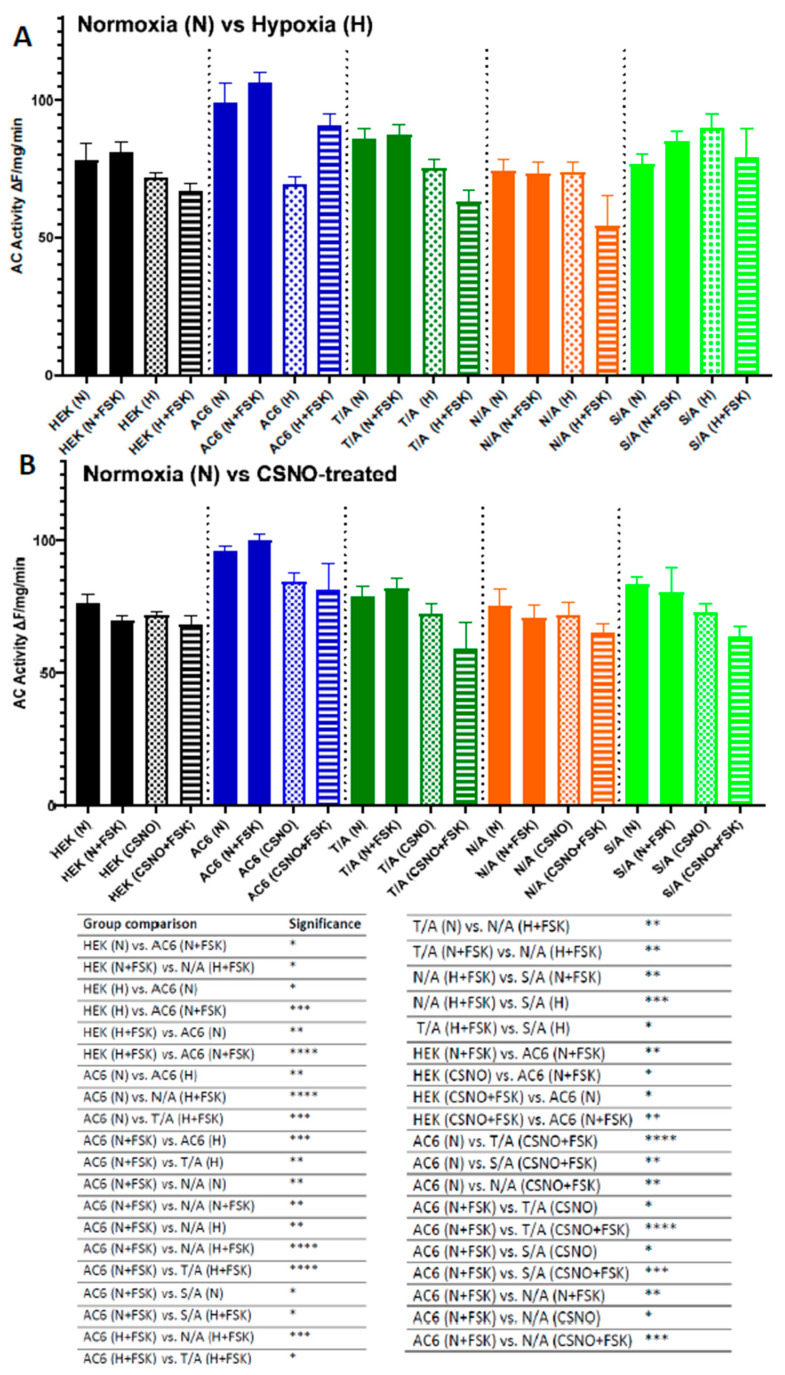
Effect of FSK site mutation on AC activity in normoxia, hypoxia, or after CSNO treatment. AC activity was measured in the total cell lysate of naïve HEK293T cells, HEK293T transfected with AC6 WT, or mutants of Forskolin interacting residues (T500A, N503A, or S1035A) in the presence of 1 mM ATP, with or without 1 µM Forskolin, following (**A**) 72 h exposure to in vitro hypoxia (H, 10% O_2_, 5% CO_2_) versus normoxic controls (N 21% O_2_, 5% CO_2_), or (**B**) 30 min treatment with 250 µM nitrosocysteine (CSNO) versus untreated controls. N = 3, One-way ANOVA with post hoc Tukey (all individual comparisons shown); * *p* < 0.05, ** *p* < 0.01; *** *p* < 0.001; **** *p* < 0.0001.

Hypoxia or CSNO treatment decreased the activity of AC6 WT and impaired its responsiveness to FSK. Among FSK site mutants, only the T/A mutation displayed conservation of hypoxia-induced impairment of basal AC activity (Figure 7A). None of the three mutants tested showed any significant change in activity upon stimulation with 1 µM FSK following hypoxia or CSNO treatment (Figure 7A,B).

### 3.4. cAMP Dynamics and FSK Interacting Mutants

**Figure 8:**
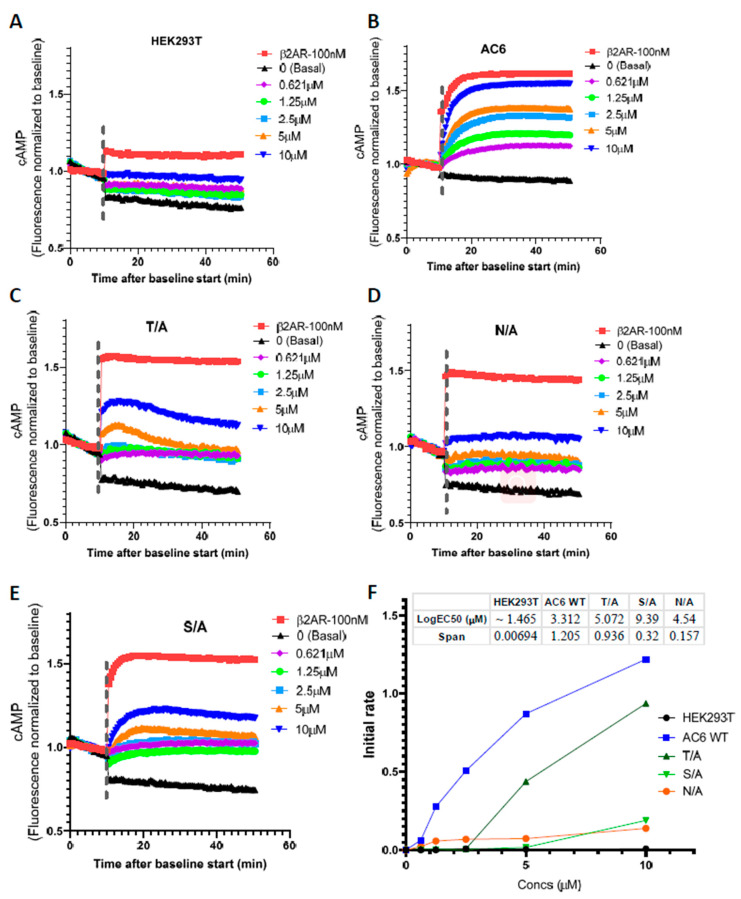
cAMP dynamics in live naïve HEK293T, or stable cell lines expressing AC6WT or FSK site mutants, using the cAMP difference detector in situ (cADDis) assay. Dose-response cAMP kinetic analysis for forskolin was performed in HEK293K cells (**A**), AC6 WT (**B**), mutants T/A (**C**), N/A (**D**), S/A (**E**). Kinetic rate constants (**F**) were derived from the shared constrained plateau of one-phase decay analysis among HEK293T, AC6 WT, and FSK binding mutants. Cells were incubated with recombinant baculovirus modified with a mammalian promoter expressing the cADDis cAMP sensor for 24 h. After baseline values were established, fluorescence decay was monitored for a 40 min time course after the addition of forskolin in a dose-dependent manner (0.621–10 µM). cAMP generation by β2AR-expressing HEK293T cells challenged with agonist isoproterenol (100 nM) used as a positive control (shown in red). Data are represented as mean ± SD, N = 3; calculated using nonlinear regression of user-defined equation for a baseline then rise to steady state time course, developed by pharmechanics.com. kτ from the one-phase decay analysis is the initial rate at a maximally effective concentration of ligand. For AC6, Kτ is equal to 1.205 normalized fluorescence units per minute.

AC generates cAMP using ATP as a substrate; total intracellular cAMP level can be reduced through AC inhibition or PDE activation, hence real-time measures of cAMP generation provide greater specificity. We, therefore, examined whether FSK site mutants become FSK unresponsive using mNeon Green-based cAMP biosensor, a cAMP Difference Detector in situ (cADDis) system in a baculovirus modified for mammalian cells (BacMam) to measure cAMP in real-time. We used the well-characterized response of HEK293T cells to Isoproterenol (10 nM) as the positive control to confirm cADDis detection. IBMX was added to each cAMP assay to inhibit PDE activity; we then measured maximum cAMP generation in a concentration-dependent manner following FSK treatment. cAMP generation by FSK site mutants was reduced as compared to WT, and there was no response to stimulation with serial concentrations (0.621–10 µM) of FSK. In the AC6 overexpressing wild-type cells, the maximal effect of forskolin-stimulated cAMP production was enhanced (Log EC_50_ = 3.312 µM). cAMP production was normalized to its control at the 10 min time point (Figure 8).

## 4. Discussion

AC catalyzes the conversion of ATP to cAMP, which regulates several physiological functions in mammals, including vascular relaxation and cardiac contraction [42]. We investigated the role of FSK interacting residues in AC catalytic activity and cAMP generation in AC isoform 6 by protein modeling and site-directed mutagenesis. We also showed how hypoxia and nitrosyl donor conditions could affect AC activity in WT AC6 compared to mutants of FSK-interacting residues.

AC isoforms in the pulmonary artery include AC3, 7, and 9, with AC6 being predominant [17]. AC5 and AC6 have high sequence similarities, while AC6 and 7 protein content can be driven by HIF response elements in their promoters [18,43]. Despite the increase in expression, we observed that the activity of the AC6 isoform is significantly inhibited under hypoxia [17]. Hypoxia may impair AC6 interaction with its key upstream activator Gαs [44] due to nitrosylation of AC6 at its interface with Gαs [22,45]. Similarly, we found nitrosyl group donor CSNO treatment decreases AC activity only in the AC6 isoform. These findings warranted understanding the structure of AC6, which may help reveal post-translational modifications and other regulatory mechanisms.

Eukaryotic ACs share a similar topology with cytosolic amino (N)- and carboxyl (C)-terminal and two repeats of six membrane-spanning helices [46]. There are two cytoplasmic domains: C_1_, which links both transmembrane domains, and C_2_ which is at the C-terminal of the protein. The C_1_ and C_2_ domains create pseudosymmetry and form the catalytic and allosteric sites. All 9 isoforms of ACs share some sequence similarities, mainly at the catalytic domain site, as well as regulatory patterns. Most previous published AC structures are chimera-based approaches [44]. Almost two decades ago, molecular structures of AC were reported to exist in three conformational states of AC: (1) catalytically inactive IIC_2_, (2) Gαs and forskolin bound IIC_2_ complexed with VC_1_, and (3) Gαs and forskolin bound IIC_2_ complexed with VC_1_, adenosine analog, and pyrophosphate [47]. Our previous homology-based molecular model of AC6 containing only catalytic domains used the PDB: 1CJK crystal structure as a template [22]. The crystal structure is unknown for any human ACs. The complete cryo-EM structure of the bovine AC9 isoform, inclusive of transmembrane regions, was reported in 2019 [30]. Using this structure (PDB: 6R3Q) as a template, we built a complete 3-dimensional structure of AC6 using a homology modeling approach. The ligand ATP analog and FSK were copied from PDB: 6R4O (another entry of AC9 cryoEM structure) and docked into the AC6 structure. Both molecules were docked in interface to both catalytic loops. FSK is independent of Gαs signaling, though the binding sites are close [48]. Homology modeling and docking revealed residues E386, F388, R1146, and K1152 that interact with AC6 substrate ATP at the catalytic site; ligand interaction analysis showed that T500, N503, and S1035 residues interact with FSK at the allosteric site. The ATP-interacting residues E386, F388, R1146, and K1152 were in proximity to the catalytic binding site that plays a role in substrate binding [28,49,50,51]. Previous studies reported that T500 and S1035 interact with the AC6 allosteric binding pocket; however, N503 was not mentioned [22,47]. N503 and S1035 residues are conserved in all the AC isoforms except AC9, where Serine 1035 aligns instead with an alanine residue.

We presumed the FSK binding pocket residues may play an essential role in AC activation and used a site-directed mutagenesis approach to dissect the mechanism of allosteric linkage with FSK. We targeted amino acids implicated in other studies to interact with FSK. The affinity of AC2 for FSK is regulated by S942 (comparable position to S1035 in the present study) which interacts with the 7-acetyl group of FSK [52]. The chimera model of VC_1_. IIC_2_ showed the close proximity of N503 residue with the FSK binding region, which may influence the binding affinity of FSK or its derivatives [9]. When these residues were mutated into alanine, the basal and FSK- stimulated AC activity decreased compared to AC6 WT. The distance of all these residues is less than 4 Å from FSK in the present AC6 molecular structure, though slightly higher in the chimera model for T500 and S1035 [22]. The superimposition of both models showed a slightly altered orientation, which could explain our finding of the additional residue, N503, as an FSK interacting residue. Detailed analysis of the FSK binding site of the T/A mutant revealed that the substitution of alanine resulted in changed orientation and loss of proximity of this residue to the docked FSK, corresponding with a large RMSD. Root mean square deviations obtained in silico have been correlated with altered protein folding, and thus may have predictive value for altered protein function [53]. It is known that small or local perturbations can produce significant RMSD changes. The magnitude of RMSD we report following a single residue substitution mutation is in line with what we have previously obtained from single-point mutation analyses [44]. Similar magnitude RMSD changes are described by others after the substitution of hydrophobic alanine for a neutral (e.g., threonine) or hydrophilic (e.g., asparagine) residue [54], and from single point mutations occurring spontaneously in the SARS-CoV2 protease that result in altered molecular dynamics [55].

Overexpression models of proteins like GPCRs have typically enhanced basal activity, which facilitates understanding of receptor-ligand interactions and the signaling pathway [56]. We confirmed stable expression with similar protein expression levels of AC isoforms 5, 6, and 7 as well as FSK binding pocket mutants by immunoblot.

Both hypoxia and CSNO significantly decreased AC6 WT activity in basal and FSK-stimulated conditions. This confirms our earlier report of decreased AC activity when pulmonary myocytes predominantly expressing AC6 were exposed to hypoxia [17], and shows that hypoxia sensitivity is a unique feature of AC6. We have previously investigated cysteines involved in the AC6- Gαs interaction; mutation of cysteine 1004 inhibits basal AC activity and cAMP generation by altering the interaction of AC6 with Gαs versus Gαi [44]. In this study, we used a similar approach focusing on FSK-dependent AC activity. Alanine substitution of T500, N503, or S1035 only mildly impairs the maximal catalytic velocity of AC6, which is understandable as these mutations impact the allosteric binding site, and not the catalytic site. The measured decrease in ATP-stimulated AC6 activity (in absence of FSK) observed from mutation of residues 500, 503, or 1035 varied between live cell and lysate preparations. T500A was most similar to WT AC6 in its ATP dose-dependent activity and hypoxia sensitivity; N503A and S1035A affected catalytic activity to a greater degree, even in absence of FSK. Due to the physical proximity of the allosteric and orthosteric binding sites as well as conformational changes imposed by Gαs binding, mutation at one site may have knock-on effects affecting protein conformation at adjacent sites, especially in whole cell preparations where the concentration of endogenous ligands cannot be controlled. This may result in an FSK site mutation altering catalytic activity at the orthosteric site. We observed a marked loss of FSK responsiveness in all three tested mutants. T/A, N/A, and S/A all showed decreased basal and FSK-stimulated AC activity in normoxic conditions. Their activity was not further impaired under hypoxic conditions or CSNO treatment. We conclude that these residues are important for FSK binding but importantly, not involved in the mechanism of hypoxic inhibition of AC6. Our data do indicate the FSK binding site of AC6 can be considered a credible drug target, as the FSK responsiveness of substitution mutants is not altered by environmental hypoxia. However, mutation of any one of these residues prevented AC6 reactivation by FSK following hypoxia or CSNO treatment. Focusing on these residues may be important when designing FSK derivatives as selective stimulators for AC6-specific activity.

To examine in greater detail the effect of FSK binding site mutations on cAMP dynamics, we used a cAMP biosensor-based monitor, to visualize cAMP fluctuations in live cells with higher temporal and spatial resolution [36,39]. Others have reported AC6 overexpression results in a higher FSK dose-response curve, while overexpressing AC2 does not [41,57]. We used the agonist challenge of a co-expressed β-adrenergic receptor as a positive control to define maximal AC-mediated cAMP generation, against which we could compare FSK responses of AC6 WT and FSK site mutants. We found reduced cAMP generation in all FSK-binding pocket mutants compared to AC6 WT. The mutants N/A and S/A showed no change in the rate constant (k) for cAMP formation in the presence of AC activator FSK dose-dependently, whereas AC6 WT indicated generation of cAMP was accelerated. T/A was only moderately impaired, still demonstrating FSK-induced acceleration. We conclude from this that N503 and S1035 are critical residues for FSK binding in AC6, while T500 may be less so.

The complete molecular structure of AC6 has presented a framework for understanding the mechanisms of its FSK-dependent stimulation in detail for the first time. The structure and biochemical analysis in this study address the physiological role of specific residues of AC6 that interact with FSK. These findings provide preliminary insight to support the design of FSK-based derivatives that would interact with AC6. Biologically important drugs act upstream of the Gαs-AC system as ligands for Gαs-coupled receptors, including adrenergic receptors. Loss of the biological activity of these agents, due to the loss of efficacy of their downstream effector enzyme, would pose a major limitation for the use of important cardiac inotropic agents and pulmonary vasodilators, particularly under conditions of tissue hypoxia. Future studies directed at synthesizing selective FSK site ligands and analyzing their functionality in cell-based assays will allow the characterization of novel drugs targeting ACs.

This study used structure-function analyses of AC6 by computational modeling and site-directed mutagenesis, to understand FSK interactions with proximal residues and their functions in accelerating enzymatic activity. Selective AC activation under hypoxic conditions is a new, exciting, unexplored pharmacology for which this study may provide the necessary direction.

## Data Availability

Publicly available protein datasets were analyzed in this study (http://www.wwpdb.org/, accessed on 20 February 2020). All other data presented in this study is available on request from the corresponding author.

## Supplementary Materials

The following supporting information can be downloaded at: https://www.mdpi.com/article/10.3390/biology12040572/s1, Figure S1: Screening cAMP responses of selected transiently transfected AC6 substitution mutants challenged with FSK.

## Abbreviations

AC	Adenylyl cyclase
cAMP	cyclic AMP
HEK293T	Human Embryonic Kidney Cells 293T
T	Threonine (THR)
N	Asparagine (ASN)
S	Serine (SER)
