# Supplementary material for: Characterization of Adenylyl Cyclase Isoform 6 Residues Interacting with Forskolin
Source: Biology (Basel). 2023 Apr 10;12(4):572. doi: 10.3390/biology12040572 (PMC10135528; doi:10.3390/biology12040572)
Supplement: Supplementary file 1 [file biology-12-00572-s001.zip › biology-2257568-supplementary.pdf]

Supplemental Figure S1: Screening cAMP responses of selected transiently transfected AC6 substitution mutants challenged with FSK

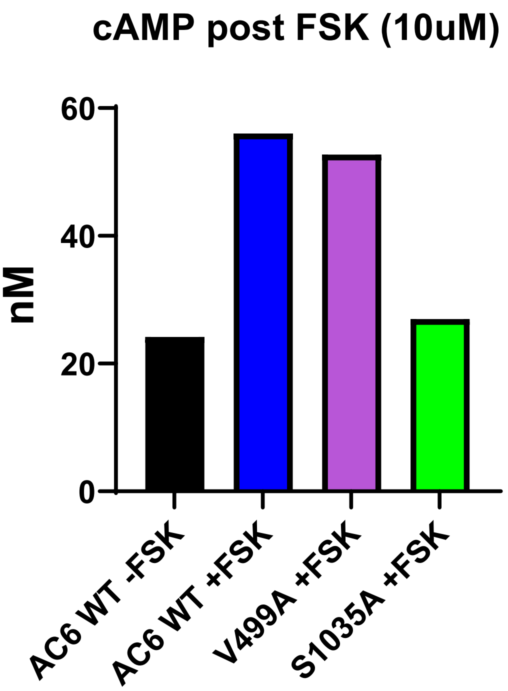

In order to preliminarily screen for residues that may be interacting with the forskolin (FSK) binding site, plasmids for AC6 WT, or AC6 alanine substitution mutants for residues including valine 499 (V499A, predicted as negative control) and serine 1035 (S1035A, predicted as FSK-interacting), were transiently transfected into HEK293T cells using 3μg DNA per $5x10^6$ cells with Lipofectamine 2000 (Life Technologies) in six-well plates. 24 hr post-transfection, cells were trypsinized and plated 20,000 cells per 96-well white transparent bottom plate, for a single-run cAMP assay in the presence or absence of 10 μM FSK treatment for 15 min, using a live cell cAMP GloMax Kit (Promega). Luminescence was measured using a FlexStation 3 microplate reader (Molecular Devices) and converted to cAMP values expressed in nM. These data are not comparable with data derived from stably transfected AC mutants, due to variability in protein expression after transient transfection, and must be considered a screening tool.
